# Clinical Utility of ‘Peekaboo Vision’ Application for Measuring Grating Acuity in Children with Down Syndrome

**DOI:** 10.22599/bioj.264

**Published:** 2022-05-04

**Authors:** Rebecca Sumalini, PremNandhini Satgunam, Ahalya Subramanian, Miriam Conway

**Affiliations:** 1 L V Prasad Eye Institute, IN; 2 City, University of London, GB

**Keywords:** Down syndrome, Grating acuity, Peekaboo Vision, Teller acuity cards II

## Abstract

Peekaboo Vision is an iPad grating acuity app built with typically developing children in mind. Given the ease of using this app in the pediatric age group, this study determined its clinical utility in children with Down syndrome. Two groups of participants (children with Down syndrome and age-matched controls) were included. Presenting binocular grating acuity was measured using Peekaboo Vision and Teller acuity cards II in random order. Parents’ feedback about their child’s engagement and time taken to complete each test was documented. Thirty-seven children with Down syndrome (males = 23; mean age = 8.1 ± 4.2 years) and 28 controls (males = 15; mean age = 8.71 ± 3.84 years) participated. Time taken to complete the tests was comparable (p = 0.83) in children with Down syndrome. Controls were significantly faster with Peekaboo Vision (p = 0.01). Mean logMAR acuities obtained with Peekaboo Vision (0.16 ± 0.34) and Teller acuity cards II (0.63 ± 0.34) were significantly different (p < 0.001) in children with Down syndrome (mean difference in acuities: –0.44 ± 0.38 logMAR (95% LoA: –1.18 to 0.3). For controls, the mean logMAR acuity with Peekaboo Vision (–0.13 ± 0.12) and Teller acuity cards II (0.12 ± 0.09) was also found to be significantly different (p < 0.001) (mean difference in acuities: –0.24 ± 0.14 logMAR (95% LoA: –0.51 to 0.03) Peekaboo Vision test can be used on children with Down syndrome. Peekaboo Vision and Teller acuity cards II can be used independently but not interchangeably. The differences in the acuity values between the two tests could be a result of the differences in the thresholding paradigms, different testing mediums and the range of acuities covered.

## Introduction

Visual acuity measurements are a useful way of screening children for refractive error and amblyopia and can also be used to quantify the effectiveness of an intervention, setting rehabilitation goals and determining eligibility/level of impairment to avail supportive benefits (National research council, 2002; Anstice and Thompson, 2014). There are a number of tests that can be used to measure visual acuity in infants and children depending on their age and cognitive ability. These tests either make use of gratings or familiar objects such as an apple/house or even letters (Verweyen, 2004). Nearly all visual acuity tests have been developed for testing typically developing children (Anstice and Thompson, 2014). Although none of the tests have been specifically developed for children with additional disabilities, tests such as the Teller acuity cards (Good, 2001), Keeler acuity cards (Clarke et al., 1997) and LEA grating paddles (Pehere and Jacob, 2019) have been adapted for testing these children, as children with poor cognitive functions are thought to respond better to grating acuity and preferential looking paradigms (Pehere and Jacob, 2019). Clinical utility can be defined in terms of testability, testing time, comparison with other testing tools, range of acuity that can be measured and ease of using the tool.

Most children with special educational needs require assessment of visual functions to understand their visual capabilities, monitor treatment effectiveness and to provide feedback to parents. Therefore, it is essential to have suitable tests for this population as well. While various acuity charts including Cardiff cards and English alphabets are used for testing visual acuity in children with Down syndrome (Zahidi et al., 2018) not all children are familiar with these optotypes and language complexity may pose a challenge in carrying out these tests, particularly when English is not their native language.

Given the advancements in digital technology, there are an increasing number of vision tests being developed and used on electronic gadgets such as computers (Ehrmann et al., 2009; Laidlaw et al., 2008), tablets (Jones et al., 2019; Jones et al., 2020; Rodriguez-Vallejo et al., 2015; Malone et al., 2014) and mobile phones (Bastawrous, 2016; Brady et al. 2015). These tests have several advantages that could potentially make them attractive for children with special educational needs, such as audio/visual feedback (Livingstone et al., 2019), accessibility and familiarity (Kabali et al., 2015). The tests can also be carried out at home or in the community, as portability is no longer an issue, thereby allowing greater versatility (Tahir et al., 2014). Test stimuli can be randomized, preventing patients from memorizing responses (Jackson and Bailey, 2004). Many digital tests are available as freeware or at a low cost, which is an added advantage compared to conventional tests which can often be expensive (Ehrmann et al., 2009).

Several digitally available tests have been found to be useful in typically developing children with and without visual impairment (Rono et al., 2018; Laidlaw et al., 2003; de Venecia et al., 2018). One such test is the Peekaboo Vision application (version 1.5) (Livingstone et al., 2019), which could potentially lend itself well to testing grating acuity in children with special educational needs, including Down syndrome. It is a freely available digital tablet-based interactive application that has been developed on an iOS platform to measure grating acuity in children. The app provides video feedback of a happy cartoon face with a ‘yippee’ sound that helps maintain attention (Livingstone et al., 2019). The app has 3 different displays of 2 (0–12 months), 4 (12–24 months) and 9 (2 years+) target presentation that can be selected based on the age of the child. Acuities obtained using the Peekaboo Vision application were found to be comparable to Keeler acuity cards in typically developing children (study 1, mean difference: 0.02 logMAR (95% LoA: 0.33 to 0.37); study 2, mean difference: 0.01 logMAR (95% LoA: –0.413 to 0.437) and the application also had a higher engagement score (study 1: p = 0.0005) (Livingstone et al., 2019). The clinical utility of Peekaboo Vision in children with special educational needs is not yet known. Given the advantages, we hypothesized that Peekaboo Vision would have good clinical utility for children with special educational needs. The main aim of this study was to determine the clinical utility of the Peekaboo Vision application in children with Down syndrome and to compare it with the commonly used Teller acuity cards (Mash and Dobson, 1998), which was noted to have comparable acuity measures as the Keeler acuity cards in typically developing children below 6 years of age (Neu and Sireteanu, 1997).

## Methods

A prospective, cross-sectional study was carried out as a part of a comprehensive health screening program organized by a non-governmental organization for children with Down syndrome in March 2019. As a part of this program, vision screening was carried out by a team of optometrists and ophthalmologists experienced in managing children with special needs. The study protocol was approved by the Institutional Review Board of L V Prasad Eye Institute (LEC: 01-19-205). The study followed the tenets of the Declaration of Helsinki. Informed written consent was obtained from parents before enrolling participants into the study.

### Participants

Parents of children less than or equal to 17 years of age with a confirmed diagnosis of Down syndrome were approached to participate in the study prior to the start of the screening process. All the parents expressed a willingness to allow their children to participate. The authors acknowledge that the normal practice for children over the age of 3 years would be to use optotypes to measure visual acuity. We are aware that VA in children with Down syndrome can be successfully measured using a variety of charts, including Teller acuity cards, Cardiff acuity test, Keeler crowded, Kay pictures crowded and single optotype acuity tests (Zahidi et al., 2018). However, the clinical experience in India has been that a majority of children or adults having Down syndrome do not respond well to optotypes. This is largely a result of unfamiliarity with these optotypes. Therefore, all participants were uniformly measured with grating acuity. This also allowed us to compare the Peekaboo Vision application with Teller acuity cards II directly. Chronologically similar aged controls with no obvious ocular conditions were also included. Control participants were recruited from a residential complex and Sunday school.

### Clinical tools

#### Peekaboo Vision application

Peekaboo Vision application (version 1.5) was used in this study on a12.9 inches (2^nd^ generation) iPad Pro with a screen resolution of 2732 × 2048. This screen size was chosen specifically as it allows for greater size and testing combinations that would be particularly useful in cases of visual impairment. The default screen brightness of 75% was used in this study (mean screen luminance: 194.9 ± 33.4 cd/m^2^ (grey scale: 214.4 ± 11.3 cd/m^2^), measured using Konica Minolta photometer (LS-110)). The iPad was switched on for at least 15 minutes prior to testing the first child in order for the screen luminance to stabilize. A uniform testing distance of 50 cm was used for all the participants (spatial frequency range: –0.18 to 1.9 logMAR). When the child’s arm length was shorter than 50 cm, or if the child did not touch the screen themselves either due to unwillingness or restricted movements of the upper limbs, the examiner touched it based on the eye movement of the child or based on the direction in which the child was pointing, either to the right or left. When the correct touch response was given, an audio (‘yippee’) along with a video (cartoon) feedback popped up, thus engaging the child and motivating them to continue the test. In the event of an incorrect touch response there was no audio or video output. Only one examiner, the first author of this study tested all the participants. This examiner has over 10 years of experience of assessing visual acuity in children with a broad range of disabilities. The examiner held the iPad Pro in a landscape orientation and was not aware of the side to which the grating was displayed. Another examiner (referred to as the observer) was constantly present and helped in holding the tape measure to ensure that the working distance was maintained during testing. Although age-appropriate alternate forced-choice paradigm has been recommended by the developer, we decided to use a uniform 2-alternate forced-choice paradigm for all children so that it could be easily compared to Teller acuity cards II; it was also convenient to track eye movements in preverbal/nonverbal children. With four targets on a small screen, it is difficult to reliably ascertain where the child is looking and could potentially introduce greater response bias. The testing was initiated for all the children from 1.9 logMAR, instead of the default 1.3 logMAR to account for the fact that some children may have severe visual impairment (Harris, 2020). Peekaboo Vision follows the staircase method of presenting gratings with a three-line logMAR down and one-line logMAR up. However, for each incorrect response the same grating was presented two more times and the response that was obtained two out of three times for that particular grating was taken as the correct response.

#### Teller acuity cards II

Teller acuity cards II were used without the testing stage as this measurement was carried out as part of a vision screening camp and using the complete set-up was not feasible. Although reducing testing distance for children with visual impairment can be carried out if needed (TAC II: reference and instruction manual, 2005), in the current study, a uniform testing distance of 55 cms was used for all children to keep it similar to the assessment carried out with Peekaboo Vision application. The length of the card (55 cms) was used as a reference to ensure that the testing distance was maintained while presenting the cards prior to commencing the test. Descending order of limits paradigm was followed to present the cards. The spatial frequency ranged from 0.32 cycles per centimeter (CPCM) to 26.0 CPCM (~ to 1.97 logMAR to 0.08 logMAR). Each card was presented twice to verify the response. If the child gave a different response for the presentation of the card, then it was presented one more time and the response that was obtained two out of three times was considered to be the final response for that particular card. In case the child was not/incorrectly responding to a particular card two out of three times, then the card that was shown earlier was considered to be the end point of the test.

### Procedure

**Figure 1:**
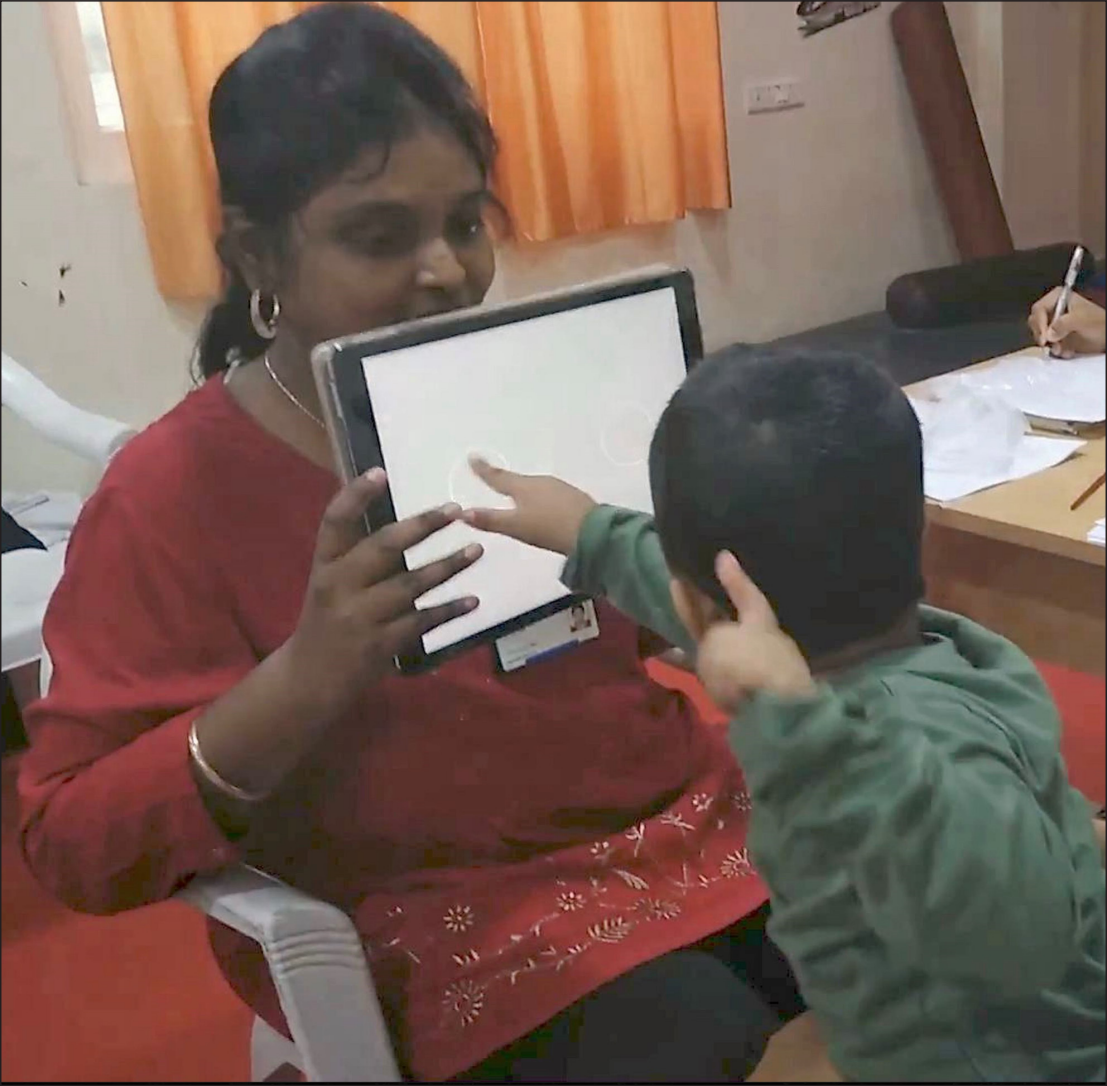
Grating acuity testing using Peekaboo Vision application.

The presenting binocular visual acuity of children with Down syndrome was measured by the examiner (***Figure 1***). The sequence of tests were randomized prior to testing using a randomly generated table in Microsoft Excel. One examiner (author RS) conducted both the tests but was masked to the stimuli. This examiner was helped by an observer who kept a record of the observations and the presented stimuli. The observer also helped in timing the test duration (using a stopwatch), handing over the charts/replacing them and in noting down the child’s responses as judged by the examiner. In addition to measuring presenting visual acuity a comprehensive vision screening was also carried out that included history taking, refraction, assessing accommodative status, anterior segment evaluation and undilated fundus evaluation (these results have not been included, as they are beyond the scope of this paper). Those children who were likely to benefit from a dilated/cycloplegic examination were referred to pediatric ophthalmologists in a tertiary eye care institute. Retest was attempted on children with Down syndrome and on controls within an average duration of 2.5 months. Verbal feedback about the child’s engagement with Peekaboo Vision application was obtained from the parents.

### Statistical analysis

Data was analyzed using IBM SPSS software (ver. 20, Chicago, USA). Paired tests were used, either parametric or non-parametric depending on the normality distribution of the outcome measure, i.e., visual acuity. p < 0.05 was considered to be statistically significant. Limits of agreement (95%) between both tests were studied using Bland-Altman analysis.

## Results

Thirty-seven children with a confirmed diagnosis of Down syndrome and a control group of 28 chronologically age-matched children with normal developmental milestones and no obvious ocular abnormalities participated (***Table 1***). Presenting visual acuity was recorded with habitual correction in eight children with Down syndrome (21.6%) and no child in the control group wore spectacles.

**Table 1:** Clinical and demographic characteristics of the participants.


S NO.	DEMOGRAPHIC/CLINICAL PARAMETER	CHILDREN WITH DOWN SYNDROME (N = 37)	CONTROL GROUP (N = 28)

1	**Age (years)**		

	(Mean ± SD)	8.1 ± 4.2	8.71 ± 3.84

	Range	1.3 to 17.0	2.3–15.0

2	**Gender (n, %)**		

	Males	23 (62%)	15(54%)

	Females	14 (38%)	13 (46%)

3	**Testing duration (Mean ± SD) in minutes**		

	Peekaboo Vision	1.8 ± 0.8	1.17 ± 0.38

	Teller acuity cards II	1.9 ± 0.8	1.44 ± 0.49

	p-value	0.83	0.01


### Down syndrome

**Figure 2:**
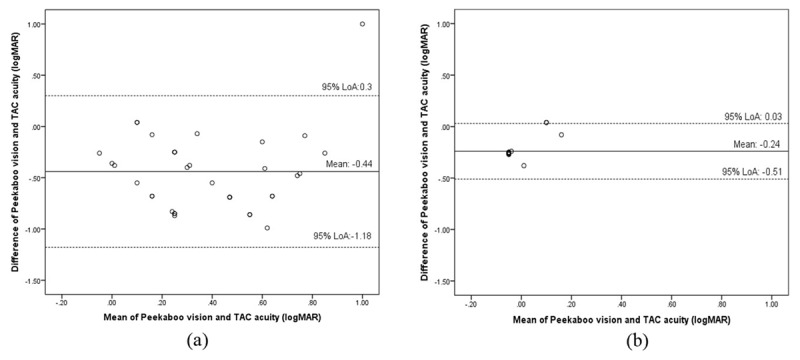
Bland-Altman plot representing 95% limits of agreement between acuity obtained using Peekaboo Vision and Teller acuity cards II in children with Down syndrome (n = 37) (2a) and in controls (n = 28) (*overlapping data points noted*) (2b).

Testability rates were high and similar for both acuity tests (Peekaboo Vision and Teller acuity cards II = 97.2%). Mean acuity obtained using Peekaboo Vision and Teller acuity cards II was 0.16 ± 0.34 logMAR (range = –0.18 to 1.5) and 0.63 ± 0.34 logMAR (range = 0.08 to 1.55) respectively. A significant difference was obtained between these two tests (p < 0.001, paired sample t-test) with a mean difference in acuities of –0.44 ± 0.38 logMAR (95% LoA: –1.18 to 0.3) (***Figure 2a***). Peekaboo Vision overestimated acuity when compared to Teller acuity cards II by approximately 4.5 lines. Time taken to complete Peekaboo Vision (mean = 1.8 ± 0.8 min) and Teller acuity cards II (mean = 1.9 ± 0.8 min) was comparable (p = 0.83, paired sample t-test) in children with Down syndrome.

### Controls

Testability rates were high for both acuity tests (Peekaboo Vision and Teller acuity cards II = 100%). Mean acuity with Peekaboo Vision and Teller acuity cards II were –0.13 ± 0.12 and 0.12 ± 0.09 logMAR respectively. A significant difference in grating acuity was obtained in controls between these two tests (p < 0.001, Wilcoxon signed-rank test) with a mean difference in acuities of –0.24 ± 0.14 logMAR (95% LoA: –0.51 to 0.03 logMAR) (***Figure 2b***). Peekaboo Vision overestimated the acuity when compared to Teller acuity cards II by approximately 2.5 lines. Significantly less testing time (p = 0.01, paired-sample t-test) with Peekaboo Vision was noted (mean = 1.17 ± 0.38 min) in comparison to Teller acuity cards II (mean = 1.44 ± 0.49 min) in the control group.

As a follow-up to the vision screening program, only a small subset of children (n = 7) visited the tertiary eye care and participated in test-retest repeatability of the VA tests, despite attempts made to reach all the children referred for further examination through follow up telephone calls. Approximately three and half lines (CR = 0.35) (1.2 octave) variability was obtained with Peekaboo Vision [mean acuity difference: 0.13 ± 0.14 logMAR, 95% LoA (limits of agreement)] and above four lines (CR = 0.43) variability with Teller acuity cards II [mean acuity difference: –0.05 ± 0.23 logMAR, 95% LoA: –0.5 to 0.4] in children with Down syndrome. Fifteen controls also underwent retest and approximately three lines (CR = 0.33) (1.1 octave) variability with Peekaboo Vision [mean acuity difference: –0.02 ± 0.18 logMAR, 95% LoA: –0.37 to 0.33] and less than one line (CR = 0.08) variability [mean acuity difference: 0.00 ± 0.05 logMAR, 95% LoA: –0.1 to 0.1,] was noted with Teller acuity cards II.

The interactive video feedback in Peekaboo Vision app was found to be a useful feature. All parents (100%) across both groups felt that the interactive feedback was helpful in maintaining their child’s attention whilst carrying out the test.

## Discussion

This is the first study to investigate the usefulness of a tablet-based, freely available application Peekaboo Vision for children with Down syndrome. Our findings suggest that there is potential to use Peekaboo Vision in measuring grating acuity in children with Down syndrome. Mean logMAR acuities obtained with Peekaboo Vision and Teller acuity cards II were found to be significantly different in children with Down syndrome (mean: –0.44 logMAR, 95% LoA: –1.18 to 0.3) and for controls (mean: –0.24logMAR, 95% LoA: –0.51 to 0.03) (p < 0.001). The present study’s control group acuity findings were comparable to the acuity differences obtained between Peekaboo Vision application and Keeler acuity cards noted in the study by Livingstone et al. (Study 2: mean difference: 0.01 logMAR, 95% LoA: –0.413 to 0.437) that was carried out in typically developing children.

Some of the differences observed between the two tests may be related to their thresholding paradigms. Teller Acuity cards II uses the descending method of limits to present stimuli and responses obtained two out of three times were used to estimate grating acuity. The procedure is manual, and the step size (0.5 octave steps) may take longer before arriving at and refining the end point. Whereas, Peekaboo Vision uses an automated staircase paradigm which may be quicker and considerably more time efficient in arriving at the end point (Spielmann et al., 2013), this was evident in the control group in our study. A shorter testing time is desirable when assessing all children particularly non/preverbal and the younger age groups given their limited attention span. The difference could also be due to the larger jump in Peekaboo Vision acuity especially while thresholding at the finer grating acuity range (i.e., an incorrect response at –0.18 logMAR will have a 0.3 logMAR jump back to 0.12 logMAR) that accounts for an absolute difference of 0.3 logMAR. Another reason could be the uniform testing distance that was used for all age groups with Teller acuity cards II and Peekaboo Vision. According to the developer’s guidelines, testing distance for Teller acuity cards II should be varied based on age (TAC II: reference and instruction manual, 2005). However, to standardize the tests, a similar testing distance was used for Teller acuity cards II and Peekaboo Vision, for all participants. Hence the highest spatial frequency that could be recorded using Teller acuity cards II in the current study was 0.08 logMAR, which could have caused an artificial ceiling effect particularly for the control group. Children with Down syndrome are noted to have hypoaccommodation. (Satgunam et al., 2019) The nature of the tests (print vs. digital) could have influenced the accommodation, differently. This was not investigated as part of this study.

High prevalence of refractive errors has been reported in children with Down syndrome (Akinci et al., 2009; Woodhouse et al., 1997). However, in the present study only 8 children with Down syndrome were noted to be spectacle users. Following the vision screening, those who needed refractive correction were prescribed spectacles and this data has not been reported here as it is beyond the scope of this paper.

Peekaboo Vision has several advantages over paper-based traditional visual acuity tests which are worthwhile to consider. It is easy to administer, is freely available and has high testability rates. Similar to Teller acuity cards II, 97% of children with Down syndrome and 100% of children in the control group were able to complete the test. It is also highly engaging, which would be particularly beneficial for children with special educational needs who tend to have a limited attention span. All parents of children who participated in the study gave positive feedback about the child’s engagement with the app. Peekaboo Vision can measure a range of acuities that would be particularly desirable on a population of children with special educational needs, who may present with a range of acuities. For example, at 50 cm, acuity measured ranges from –0.18 to 1.9 logMAR. By alternating the working distance, the range can be further expanded to –0.18 to 2.11 logMAR. In addition, as Peekaboo Vision application has an automated threshold, it is easier for even a novice examiner to carry out the test as in comparison to the experience that is often recommended to perform the test using conventional paper-based cards (Getz et al., 1996). However, this may be challenging if an inexperienced examiner has to judge responses based on the eye movements of the child and ‘touch’ the screen for the child. Good eye-hand coordination is needed to perform the test using the Peekaboo Vision application. Children with special educational needs (e.g., with cerebral palsy) may have limited eye-hand coordination, which would make the task challenging. In such cases, the examiner should be able to judge the eye responses and touch the grating on behalf of the child.

Test-retest repeatability is an important measure to determine the clinical validity of any test (Sanchez and Binkowitz, 1999). Repeatability was noted to be within 1 octave (i.e., doubling/halving of the spatial frequency) using acuity card procedures (Mackie and McCulloch, 1995) in several studies in children with special educational needs, such as cerebral palsy (76%) (Hertz and Rosenberg, 1988), Down syndrome (73%) (Hertz, 1987), and other neurological conditions (88%) (Getz et al., 1996). A study by Livingstone et al. in 2019 on typically developing children using Peekaboo Vision reported approximately three lines variability in both studies, i.e., in Malawi and the United Kingdom (study 1: 95% LoA: –0.283 to 0.198 logMAR, CR = 0.27; study 2: 95% LoA: –0.344 to 0.320 logMAR, CR = 0.32), which corresponds to less than 1 octave and 1.1 octave respectively. This was comparable to the present study in controls. Due to poor follow-up, only a small number of children with Down syndrome were recruited for a retest in this study which is a limitation.

The clinical testing of the Peekaboo Vision app in children with Down syndrome reveals comparable testing time similar to the well-established Teller acuity cards II and significantly shorter time in controls. In addition to the descending method of limits paradigm used for thresholding acuity using Teller acuity cards II, the mechanical shifting of the cards could also account for the longer testing time. A larger sample size would be needed to determine the test-retest repeatability of Peekaboo Vision in children with Down syndrome and other disabilities. This would not only prove useful in the regular clinical testing of children with disabilities but also to quantify the true effect of any intervention using grating acuity.
